# Microbial metabolites are involved in tumorigenesis and development by regulating immune responses

**DOI:** 10.3389/fimmu.2023.1290414

**Published:** 2023-12-19

**Authors:** Jiahui Liu, Ruxian Tian, Caiyu Sun, Ying Guo, Lei Dong, Yumei Li, Xicheng Song

**Affiliations:** ^1^Department of Otorhinolaryngology, Head and Neck Surgery, Yantai Yuhuangding Hospital, Qingdao University, Yantai, China; ^2^Shandong Provincial Clinical Research Center for Otorhinolaryngologic Diseases, Yantai Yuhuangding Hospital, Qingdao University, Yantai, China; ^3^Yantai Key Laboratory of Otorhinolaryngologic Diseases, Yantai Yuhuangding Hospital, Qingdao University, Yantai, China

**Keywords:** microbiome, microbial metabolites, host signaling pathway, immune responses, tumorigenesis and development microbiome, tumorigenesis and development

## Abstract

The human microbiota is symbiotic with the host and can create a variety of metabolites. Under normal conditions, microbial metabolites can regulate host immune function and eliminate abnormal cells in a timely manner. However, when metabolite production is abnormal, the host immune system might be unable to identify and get rid of tumor cells at the early stage of carcinogenesis, which results in tumor development. The mechanisms by which intestinal microbial metabolites, including short-chain fatty acids (SCFAs), microbial tryptophan catabolites (MTCs), polyamines (PAs), hydrogen sulfide, and secondary bile acids, are involved in tumorigenesis and development by regulating immune responses are summarized in this review. SCFAs and MTCs can prevent cancer by altering the expression of enzymes and epigenetic modifications in both immune cells and intestinal epithelial cells. MTCs can also stimulate immune cell receptors to inhibit the growth and metastasis of the host cancer. SCFAs, MTCs, bacterial hydrogen sulfide and secondary bile acids can control mucosal immunity to influence the occurrence and growth of tumors. Additionally, SCFAs, MTCs, PAs and bacterial hydrogen sulfide can also affect the anti-tumor immune response in tumor therapy by regulating the function of immune cells. Microbial metabolites have a good application prospect in the clinical diagnosis and treatment of tumors, and our review provides a good basis for related research.

## 1 Introduction

All the microbiota associated with the human body, including small eukaryotes, fungi, archaea, bacteria and viruses are referred to as the human microbiome, which affects human health and diseases (1). Recognized as the second leading cause of death in the world, cancer is a major threat to human health (1), and 20% of human malignancies have been found to be associated with dysregulation of the human microbiome (2). In general, the microbiome rarely directly causes cancer. However, they can be complicit in cancer growth by regulating immunity, such as affecting the activity of primary and secondary lymphoid tissues, secreting cytokines that initiate Toll-like receptors (TLRs), producing microbial metabolites, and performing antigen mimicry with cancer cells (3). Human anatomical niches such as conjunctiva, nose, oral cavity, gut, vagina and skin are all endowed with unique and functionally relevant microbial populations (4), which are numerous and extremely active, and can produce a large number of metabolites (**Table 1**). Metabolomics, which centers on the study of metabolites in biological systems and analyzes samples using mass spectrometry-based techniques, is a relatively new field of research (13). Thanks to the development of metabolomics, the role of microbial metabolites in tumor has been increasingly characterized.

**Table 1:** The microbial metabolites produced by the microbiota.

Metabolite	Substrate	Producers	References
Short-chain fatty acid	dietary fiber, starch	*Firmicutes* including *Eubacterium hallii*, *Eubacterium rectale* and *Faecalibacterium prausnitzii, Clostridium, Bifidobacterium, Propionibacterium, Bacteroides*, and *Lactobacillus*	(5, 6)
Microbial tryptophan catabolites	tryptophan	*Bacteroides, Escherichia coli*, and *Clostridium*	(7, 8)
Polyamine	arginine, ornithine	*Acinetobacteria, Bacteroides, Firmicutes, Fusobacteria*, and *Proteobacteria phyla*	(4, 6, 9)
Hydrogen sulfide	methionine, cysteine	Anaerobic sulfate-reducing bacteria (SRB) such as *Desulfobacter, Escherichia coli, Staphylococcus aureus, Salmonella thyphimurium, Helicobacter pylori, Fusobacterium nucleatum, various Clostridium species, Enterobacter* and *Klebsiella spp*	(10, 11)
Secondary bile acids	cholesterol	*Bacteroides, Lactobacillus, Bifidobacterium, Clostridium (clusters XIVa* and *XI)*, and *Eubacterium*	(7, 9, 12)

### 1.1 Intestinal microbial metabolites and tumor

Studies on microbial metabolites in human blood and urine have revealed a strong correlation between the production of microbial metabolites and human tumors. If the production of metabolites becomes abnormal, it can potentially impact tumor formation and progression (9). It is important to note that although the microbiota in various anatomical locations within the human body can synthesize multiple metabolites, the intestinal microbiota is unique in terms of metabolite production. This is because the intestinal microbiota represents the largest density and absolute abundance of the microbiome in the human body (14) and produces approximately 10% of metabolites in the blood and over 50% of metabolites in stool and urine (7). Intestinal microbiota and its metabolites not only impact the balance of the intestine and its surrounding areas (15), but they can also travel to other parts of the body via the bloodstream. They can influence inflammation and the development of tumors in specific organs through pathways such as the gut-brain axis (16–18), gut-lung axis (19, 20), and gut-hepatic axis (21, 22). In the human body, short-chain fatty acids (SCFAs), microbial tryptophan catabolites (MTCs), polyamines (PAs), hydrogen sulfide and secondary bile acids are the primary metabolites produced by the intestinal microbiota through the metabolism of the three main undigested nutrients, namely carbohydrate, protein and fat are the three major nutrients in the diet. Compared with other microbial metabolites, such as vitamin and histidine, SCFAs, MTCs, PAs, hydrogen sulfide and secondary bile acids have significant impacts on tumor development (1, 14, 23). Numerous studies have demonstrated their involvement in the occurrence and development of colorectal inflammation and tumors (24–30). Additionally, the role of SCFAs in lung cancer (31), PAs in brain tumors and neuroblastoma (32, 33), SCFAs, MTCs and secondary bile acids in digestive system tumors (21, 34–36), and SCFAs, MTCs and PAs in gynecological tumors and melanoma (31, 34, 37–42) have also been confirmed. Therefore, this review focuses on the above five intestinal microbial metabolites.

### 1.2 Immune cells and tumor

**Figure 1:**
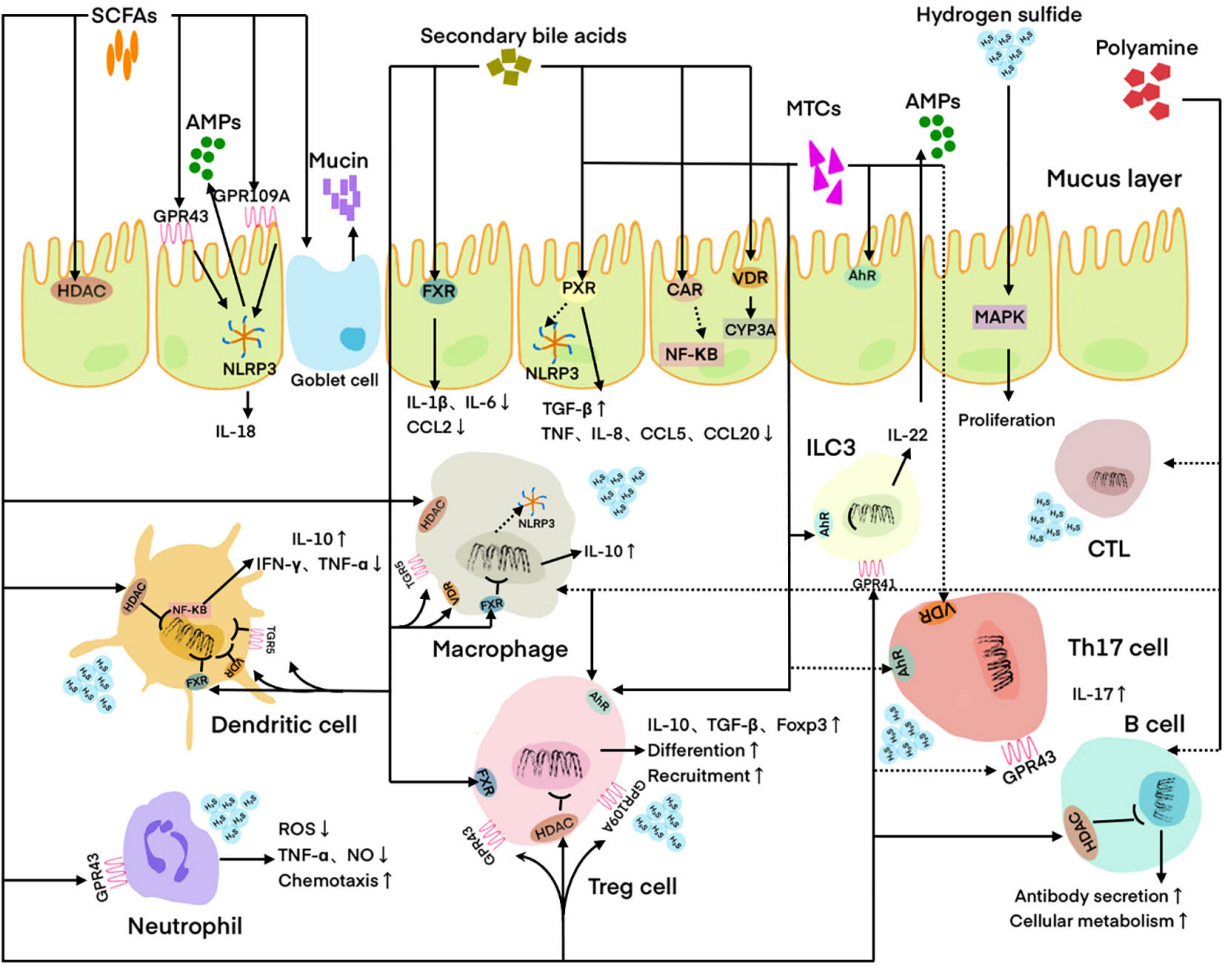
Regulation of host immune cells and IECs by microbial metabolites. DC is regulated by SCFAs, hydrogen sulfide, and secondary bile acids. Neutrophil is regulated by SCFAs and hydrogen sulfide. Macrophage is regulated by SCFAs, PAs, hydrogen sulfide, and secondary bile acids. Treg cell is regulated by SCFAs, MTCs, polyamines, and secondary bile acids. Th17 cell is regulated by SCFAs, MTCs, and secondary bile acids. CTL is regulated by PAs. ILC3 is regulated by MTCs. B cell is regulated by SCFAs and PAs. IEC is regulated by SCFAs, MTCs, hydrogen sulfide, and secondary bile acids. Solid lines indicate facilitation and dashed lines indicate inhibition. SCFAs, Short-chain fatty acids; MTCs, Microbial tryptophan catabolites; Pas, polyamines; AMPs, Antimicrobial peptides; HADC, Histone deacetylase; FXR, Farnesoid X receptor; PXR, Pregnane X receptor; CAR, Constitutive androstane receptor; VDR, Vitamin D receptor; AhR, Aryl hydrocarbon receptor; MAPK, Mitogen-activated protein kinase; ILC3, Innate lymphoid cell 3; CTL, Cytotoxic T lymphocyte; IEC, Intestinal epithelial cell; ROS, Reactive oxygen species.

Tumor cells shape a complex and evolving tumor microenvironment (TME) by secreting various factors that influence the surrounding stroma. Adaptive immune cells, such as T cells and B cells, and innate immune cells, such as dendritic cells (DCs), macrophages, neutrophils, myeloid-derived suppressor cells (MDSCs), innate lymphoid cells (ILCs) and natural killer (NK) cells, are important components of TME (43). The anti-tumor immune response of the body relies heavily on adaptive immune cells, particularly T cells (44). The absence of CD8+ T cells results in immune response dysfunction within the TME (45). Regulatory T (Treg) cells, a subset of CD4+ T cells characterized by the expression of the transcription factor FOXP3, exert a potent inhibitory effect on tumor immunity (46). The second group of adaptive immune cells in TME are B cells, which are capable of producing cytokines that directly or act on T cells to influence anti-tumor immune responses (47). Among innate immune cells, DCs (48), neutrophils and their subtype MDSCs (44, 49, 50) and ILCs (51) can mediate the cross-priming of tumor-specific T cells, macrophages can phagize pathogens and tumor microenvironment products (52), and NK cells (53) can exert cytotoxic effects, to initiate and maintain anti-tumor immunity. Immune cells are involved in maintaining a healthy microbial community, and microbiota can also regulate the function of immune cells by producing metabolites (54). For example, the Th17/Treg balance is critical in cancer progression, and excessive inflammation caused by Th17 cells or excessive immune suppression induced by Treg cells may lead to carcinogenesis (46). SCFAs (55), MTCs (56), and secondary bile acids (57) produced by microbiota can increase the number of Th17 cells and reduce the number of Treg cells playing a role in suppressing cancer, while hydrogen sulfide (58) produced by the microbiota can affect the balance to promote cancer (**Figure 1**).

### 1.3 Microbial metabolites, immune responses and tumors

Microbial metabolites are crucial molecular signals within the host that contribute to the maintenance of immune homeostasis (5). Smith K et al. observed that germ-free mice raised in a microbe-free environment had significantly underdeveloped immune systems, demonstrating the important role of the microbiota in immune function (59). These metabolites can participate in the regulation of immune cell development and differentiation (60), modulate the balance and function of innate and adaptive immune cells through direct and indirect mechanisms, and influence the occurrence and development of tumors (61). SCFAs, MTCs, PAs, hydrogen sulfide and secondary bile acids produced by intestinal microbial metabolism can affect the function of immune cells, such as T cells, B cells, DCs, macrophages, neutrophils, MDSCs and ILCs by activating cell receptors, regulating enzyme expression and influencing cell differentiation (**Table 2**). In addition, microbial metabolites can affect the function of intestinal epithelial cells (IECs) and participate in the regulation of mucosal immunity. Mucosal immunity is an important part of the human immune system. In the intestinal mucosa, immune cells cooperate with IECs to strengthen the intestinal mucosal barrier function (118, 119). The altered availability of metabolites and regulation of the immune system are the primary mechanisms through which microbial-derived metabolites impact tumor development (1).

**Table 2:** Regulation of host immune system by microbial metabolites.

Microbial metabolites	Mechanism	Immune cells	Effects on the immune system	References
**Short-chain fatty acid**	Direct inhibition of HDAC	monocytes, neutrophils, macrophages, bone marrow stem cells, CD4+ T cells, IEC	Leads to NF-κB inactivation and down-regulation of proinflammatory cytokine TNF-α, NO, IL-6 and IL-12 production. Blocks the differentiation of bone marrow stem cells into DCs. Promotes the differentiation of CD4+T cells into Treg cells. Promotes IECs produce retinoic acid.	(62–68)
	Activation of GPR43	neutrophil, Treg cell, Th17 cell, CD8+ T cell, DC, IEC	Induces neutrophils chemotaxis and decreases the production of ROS, TNF-α and NO. Promotes CD8+T cell exhaustion. Over activates DCs and causes them to die. Increases the Treg cells. Reduces the Th17 cells. Increases the production of AMPs.	(55, 69–71)
	Activation of GPR41	CD4+ T cell, ILC, IEC,	Increases the production of IL-22. Increases the production of AMPs and promotes epithelial barrier function.	(72, 73)
	Activation of GPR109A	macrophages, DCs, Treg cell, T cell	Increases the production of anti-inflammatory effector molecules and IL-10.	(74)
	Effects on the efficacy of checkpoint inhibition therapy	T cell, DC, Treg cell,	Regulates the differentiation of T cells into effector or Treg cells. Reduces the body’s response to IL-2.	(40, 75)
	Effects on the efficacy of adoptive cell therapy	CD8+ CTL	Increases the production of effector molecules such as CD25, IFN-γ, and TNF-α.	(34)
	Effects on the efficacy of radiotherapy	DC, CD8+T cell	Increases DCs antigen presentation and primes CD8+T cells.	(31)
**Microbial tryptophan catabolites**	Activation of AhR	Treg cell, Th17 cell, ILC3, Tumor-infiltrating lymphocyte	Increases the Treg cells. Reduces the Th17 cells. Increases the production of IL-22. Promotes lymphocyte infiltration to tumors.	(25, 56, 76–78)
	Activation of PXR	IEC, Tumor-infiltrating lymphocytes	Maintains intestinal epithelial barrier structure and function. Promotes lymphocyte infiltration to tumors.	(78, 79)
	Effects on host IDO1 expression	DC, T cell, Treg cell	Mediates T cell anergy and enhances Treg function through IDO-expressing DCs.	(80–88)
	Epigenetic modifications	DC, CD8+T cell	Promotes IL12a production in DCs by enhancing H3K27ac binding at the IL12a enhancer regions. Enhance the function of tumor-infiltrating CD8+ T cells by changing chromatin accessibility.	(89)
	Effects on MPO	neutrophil, PMN	Activation of neutrophils MPO enhances the efficacy of the chemotherapeutic agent FIRINOX for mPDAC. Inhibits MPO activity of PMN to prevent inflammation and reduce bystander tissue damage.	(36, 90)
**Polyamine**	Effects on antitumor immune response	B cell, T cell, Tumor-infiltrating immunosuppressive myeloid cell population	Induces the activation of B and T cells. Induces cytotoxic activity and T cell proliferation. Leads to immunosuppression of the tumor microenvironment. Reduces the expression of adhesion molecules such as CD44 and LFA-1. Reduces the production of cytokines such as IFN-γ and TNF.	(91–99)
**Hydrogen sulfide**	Regulation of intestinal mucosal immunity	IEC, T cell	Has a bell-shaped effect that is protective of the intestinal epithelium at low concentrations and harmful at high concentrations. Upregulates cytokine production and cellular activation of the Th17 and Treg profiles in mesenteric lymph node T cells. Induces excessive proliferation of the colonic mucosa.	(26, 58, 100)
	Protection of the bacteria itself	neutrophil	Regulates the function of host neutrophils. Upregulates the bacteria’s antioxidant defense mechanisms.	(101, 102)
	Regulation the composition and function of immune cells	CD11b+ leukocyte, B cell, T cell, Treg cell, Th17 cell	Increases the number of CD11b+ leukocytes, B cells, CD8+ T cells, and Treg cells. Trigger Th17 cell-mediated inflammatory response. Activates anti-tumor immune responses.	(58, 103)
**Secondary bile acids**	Activation of TGR5	peripheral blood monocytes, bone-marrow-derived macrophage, bone-marrow-derived DC	Inhibits M1 secretion of proinflammatory cytokines such as TNF-α, IFN-γ, IL-6, and IL-12. Promotes the differentiation of M2 macrophage secreting IL-10. Blocks the activation of NLRP3 inflammasome.	(29, 104, 105)
	Activation of FXR	IEC, CD14+ monocyte, DC, macrophage, Treg cell	Regulates IECs integrity and microbial composition. Reduces the expression of proinflammatory cytokines such as IL-1β and IL-6 and chemokines such as CCL2. Restricts TLR4 -dependent proinflammatory cytokine expression. Inhibits NLRP3 inflammasome activation. Increases serum IL-10 levels, perpetuates splenic DCs and increases the number of Treg cells.	(106–110)
	Activation of PXR and CAR	IEC	Promotes the expression of TGFβ. Restricts the expression of TNF, IL-8, CCL5, and CCL20. Inhibits TLR4-dependent proinflammatory cytokine production. Inhibits the activation of NF-κB and the expression of DME and promotes the detoxification and clearance of bile acids.	(111–114)
	Activation of VDR	macrophage, DC, Treg cell, IEC, Th1 cell, Th17 cell	Inhibits macrophage activation from monocytes. Inhibits DC maturation. Promotes Treg cell differentiation. Inhibits proinflammatory Th1 and Th17 responses. Inhibits the expression of proinflammatory cytokines in IECs.	(57, 115, 116)
	Regulate the level of chemokine CXCL16 in LSECs	CXCR6+ liver NKT cell	Affects the production of IFN-γ. Changes the selective tumor suppressor effect of the liver. Affects the growth of liver cancer.	(21, 117)

However, on the one hand, the specific mechanisms by which microbial metabolites exert their effects have not been fully characterized. On the other hand, the interindividual differences of metabolites and their effects at different concentrations also need to be further explored. Based on the existing studies and reports, we review five microbial metabolites that are involved in the process of tumorigenesis and development by regulating the immune response. Our aim is to establish a connection between microbial metabolites and tumors through the immune response and provide a reference for studying the potential mechanisms of microbial metabolites in tumorigenesis and development, as well as discovering new strategies for tumor immunotherapy.

## 2 SCFAs

SCFAs have a skeleton of 1-6 carbon atoms and mainly include acetic acid, propionic acid and butyric acid. They are volatile fatty acids produced by colonic bacteria fermenting starch and dietary fiber that is not absorbed in the small intestine (120). There are many anaerobic microorganisms involved in SCFAs production in the colon, including some bacteria of the phylum *Firmicutes, Bifidobacterium, Lactobacillus, Bacteroides, Propionibacterium* and *Clostridium* (6). Among them, acetic acid and propionic acid are mainly from *Bacteroides*, which are taken up by the blood and distributed to other tissues and organs, and butyric acid is mostly produced by some highly abundant species of *Firmicutes*, such as *Eubacterium hallii, Eubacterium rectale* and *Faecalibacterium prausnitzii*, which is produced as an energy source for IECs and affect metabolism (121, 122).

### 2.1 SCFA directly or indirectly inhibits HDAC to regulate the immune system

HDAC is an enzyme that can mediate the deacetylation of histones, an epigenetic modification in the host (123). Inhibition of HDAC in immune cells, such as monocytes, neutrophils, macrophages, bone marrow stem cells and CD4+ T cells, can affect cytokine production and cell differentiation to suppress inflammation and cancer. SCFAs propionate and butyrate can inhibit HDAC resulting in increased histone acetylation to play an anti-inflammatory and anti-cancer role, while acetate has no such effect (124). Usami M et al. stimulated peripheral blood mononuclear cells with propionate and butyrate and found that they could inhibit HDAC to inhibit the production of the proinflammatory cytokine TNF-α by secreting PGE2 and down-regulate the activation of the pro-inflammatory NF-κB pathway induced by lipopolysaccharide (LPS) (62). Similarly, Vinolo MA et al. stimulated neutrophils with propionate and butyrate and found that they also can reduce the production of proinflammatory TNF-α, CIN-2αβ, and NO and inhibited LPS-induced NF-κB activation by inhibiting HDAC. Oral administration of butyrate precursors to rats reduced the recruitment of neutrophils to sites of inflammation, also demonstrating the anti-inflammatory effect of butyrate (63). In addition, Chang PV et al. used n-butyrate to stimulate bone marrow-derived macrophages and found that n-butyrate also can inhibit macrophage HDAC, leading to the down-regulation of LPS-induced proinflammatory NO, IL-6 and IL-12 (64). In colitis mouse models, Singh N et al. found that propionate and butyrate can inhibit HDAC in bone marrow stem cells, depending on the Na (+)-coupled monocarboxylate transporter SLC5A8 that transport them into cells, to block differentiation into DCs, resulting in the inability to present antigens to T cells and initiate adaptive immune responses (65). *In vivo* and *in vitro* experiments also showed that propionate and butyrate can inhibit HDAC in CD4+ T cells, which can enhance the Foxp3 acetylation, to induce extrathymic differentiation of Treg cells, dependent on CNSI, an intronic enhancer required for extrathymic Treg cells differentiation but not for thymic differentiation, promoting IL-10 production to reduce intestinal inflammation (66, 67). Furthermore, Mowat C et al. recently used propionate and butyrate to stimulate colon cancer cells *in vitro*. They found that they could inhibit HDAC, promoting chromosome deacetylation to induce DNA damage and escape to the cytoplasm. Then cGAS/STING pathway was activated to stimulate IFN-γ production and up-regulate MHCI expression, promoting the activation of CD8+ T cells to kill tumor cells. These results were confirmed in organoids derived from colon cancer patients and in mouse models of colon cancer (125).

SCFAs are also ligands of GPCRs, which can indirectly inhibit HDAC through a GPCRs-dependent mechanism (126). GPCRs, including GPR43, GPR41 and GPR109A, are expressed in a variety of cells including IECs and immune cells. They are essential signaling pathways for SCFAs to achieve many regulatory functions. First, the expression of GPR43 in host cells is a chemotactic surface receptor that controls the signaling of microbiota-derived SCFAs through intracellular Ca2+ signaling to induce host neutrophil chemotaxis (69). In addition, GPR43-deficient mice with chronic inflammation had more Th17 cells and fewer Treg cells in the intestines than wild-type mice after propionate treatment. They also had shorter colons and more severe inflammation. This suggests that propionate inhibits Th17-driven chronic intestinal inflammation and colon cancer development through GPR43 (55). Lavoie S et al. stimulated the colitis mouse models with SCFA and found that mice with conditional disruption of GPR43 in DCs developed significantly more spontaneous colon tumors. On the one hand, it can over-activate DCs by promoting CD80 expression to increase their death. On the other hand, it can promote the production of IL-27+ DCs through TLR 4, IFN-γ and NF-κB signaling pathways, increasing the expression of co-inhibitory molecules such as CD 39, PD-1 and LAG-3 in CD8+ T cells, which can promote the exhaustion of CD8+ T cells. Both of them can suppress anti-tumor immunity to promote colon tumorigenesis (70). Second, GPR41 is required for microbiota-derived SCFAs to inhibit HDAC and promote IL-22 production by host CD4+ T cells and ILCs (72). Most IL-22-related molecules are encoded by inflammatory bowel disease (IBD) susceptibility genes (127) and play a central role in host resistance to intestinal inflammatory injury by maintaining the integrity of the epithelial barrier and inducing the production of antimicrobial peptides (AMPs) (73). Supplementation with SCFAs can increase IL-22 production to reduce intestinal inflammation and tumor susceptibility. Third, GPR109A on colonic macrophages and DCs can be activated by microbiota-derived niacin and butyric acid to induce the production of IL-10, which protects against colitis and colon cancer by inducing the differentiation of IL-10-producing T cells and Treg cells and increasing the amount of monocyte-derived anti-inflammatory effector molecules (74).

For IECs, SCFA butyrate can inhibit HDAC to mediate the acetylation of SLC5A8 promoter region, stimulating the expression of SLC5A8. Then the intracellular uptake of butyrate is enhanced, upregulating aldh1a1 or aldh1a3 transcriptional expression to increase retinoic acid conversion, which can maintain epithelial homeostasis (68). microbiota-derived SCFAs can also bind to GPR43 to activate mTOR/STAT3, inducing the production of AMPs (71). In addition, SCFAs can bind GPR43 of IECs to stimulate K (+) efflux and hyperpolarization, leading to NLRP3 inflammasome activation (128). In the intestinal epithelium, butyrate can also promote the production of IL-18, a cytokine located downstream of NLRP3 that can inhibit colon tumors, in a GPR109A-dependent manner (74, 129). Overall, SCFAs can act on IECs to strengthen the integrity of the intestinal barrier.

### 2.2 SCFAs modulate immune responses in cancer therapy

Checkpoint inhibition therapy is a new direction of cancer immunotherapy, and changes in metabolism, endocrine regulation and environment *in vivo* can affect its efficacy. For example, in clinical practice, ipilimumab (anti-CTLA-4) is approved alone or in combination with nivolumab (anti-PD-1) for the treatment of metastatic melanoma (MM) (41). Studies have shown that Treg cells located in the tumor microenvironment are necessary to improve the efficacy of checkpoint inhibitors. However, their immunosuppressive activity also weakens the effect of cancer immunotherapy. Therefore, Treg cells may become targets for future cancer treatments (130). SCFAs are considered to be key microbial metabolites in T cell homeostasis, which can regulate the differentiation of T cells into effector or Treg cells (75). For example, in mice and MM patients, high propionic acid and butyric acid levels can reduce the efficacy of anti-CTLA-4 treatment. Because they lead to an increased proportion of Treg cells, decreased activation of DCs and effector T cells, and reduced response to IL-2, leading to resistance to CTLA-4 blocking (40).

An alternative cancer immunotherapy modality is adoptive cell therapy with tumor-specific CD8+ CTLs. CTLs are generated from endogenous lineages or by genetic engineering with a chimeric antigen receptor (CAR) or T cell receptor (TCR). Genetic modification of the CAR confers a novel antigen-specific target on T cells and kills tumor cells. One of the targets of CAR T cells is the orphan receptor ROR1, which is expressed in many tumors of epithelial origin (131). Research on mice suggests that the intestinal microbiota can produce valeric acid and butyric acid, through epigenetic modifications and metabolism, to increase the content of effector molecules *in vivo*, including IFN-γ, CD25 and TNF-α. In isogenic mouse models of pancreatic cancer and melanoma, they can significantly enhance the anti-tumor activity of antigen-specific CTL and ROR1-CAR T cells, suggesting that microbiota-derived SCFAs may be possible to optimize adoptive T cell therapy for human cancers (34).

In addition, using mouse models of melanoma and lung cancer, Uribe-Herranz M. et al. showed that the elimination of *Clostridium*-derived propionic acid and butyrate with vancomycin, which exerts immunosuppressive effects *in vivo*, can enhance the efficacy of tumor radiotherapy by activating CD8+T cells and enhancing DC-mediated antigen presentation (31). Guo et al. gave specific pathogen-free (SPF) mice a high dose of total body radiation (9.2 Gy), and conducted microbiota adoptive transfer experiments and monoassociation studies. Metabolomics revealed that the presence of intestinal microbiota-derived propionic acid in radiation models was associated with radioprotection and reduced pro-inflammatory responses (132).

In conclusion, we reviewed the anti-inflammatory and anti-cancer effects of microbiota-derived SCFAs by regulating the functions of immune cells and IECs. On the one hand, SCFAs can inhibit HDAC of immune cells, such as neutrophils, macrophages, T cells, or act as ligands for GPCRs, to affect the production of cytokines and induce cell differentiation, playing anti-inflammatory and anti-cancer effects. On the other hand, for IECs, SCFAs can also inhibit HDAC or act as ligands for GPCRs to play a protective role in colorectal inflammation and cancer by affecting the production of cytokines, retinoic acid and antimicrobial peptides. In addition, SCFAs can also enhance the effect of cancer immunotherapy by regulating the phenotype and function of T cells.

## 3 MTCs

As one of the essential amino acids in the human body, tryptophan is metabolized mainly through kynurenine (Kyn) pathway, tryptamine pathway, 5-hydroxytryptamine pathway and indole pathway (8). Approximately 4-6% of the tryptophan in the body is directly utilized in the gastrointestinal tract by intestinal bacteria, which results in the availability of tryptophan for the host being partially limited. Tryptophan is metabolized by intestinal bacteria via the indole pathway to a variety of metabolites with functions that regulate the host immune system (133). For example, *Bacteroides* spp. and Escherichia coli can convert tryptophan into tryptamine and indole (134), *Clostridia* can decompose tryptophan into indole pyruvic acid and then into indole-3-acetic acid (3-IAA) (135), and *Lactobacillus reuteri* and *Lactobacillus johnsonii* can convert tryptophan into indole-3-aldehyde (I3A) (136). The most important of the numerous MTCs are tryptamine, indole acid derivatives and indole. These substances primarily regulate the immune system by activating receptors of immune cells, affecting the enzyme activity within the host body and epigenetic modification. As a result, they can influence the occurrence and development of tumors and the immune response in cancer therapy.

### 3.1 MTCs affect the immune system by activating cellular receptors

Aryl hydrocarbon receptor (AhR) is a ligand-dependent transcription factor expressed by cells of the immune system (137) and many types of human tumors, including urothelial, prostate, breast, ovarian, gastric, liver, pancreatic, lung, medulloblastoma, glioma and T-cell leukemia (138, 139). Studies have shown that AhR activation can alter both innate and adaptive immunity *in vivo* (140–142). There is evidence that AhR is involved in cancer initiation and metastasis (138). Analysis of human fecal samples by Sun et al. revealed lower levels of indole in the fecal samples of colorectal cancer patients compared to healthy individuals (25). Busbee PB et al. found that Indole-3-Carbinol (I3C) could activate AhR in colitis mice, resulting in the decrease of Th17 cells, which are related to the proinflammatory response leading to colitis, and the increase of Treg cells, which are related to maintaining intestinal homeostasis, leading to the attenuation of inflammation. I3C also increased the differentiation of IL-22-producing ILC3 cells (56). As mentioned earlier, IL-22 plays a key role in intestinal homeostasis. RORγt is a nuclear receptor that plays a key role in the development of ILC3 (143). In the gut, depending on AhR, RORγt+ ILCs are the major producers of IL-22 and AhR and RoRγt can interact. On the one hand, IL-7, which is required for RORγt+ ILCs survival, will be reduced in the large intestine of mice in the absence of AhR, leading to an increased apoptotic rate of RORγt+ ILCs and decreased IL-22 production. On the other hand, RoRγt can significantly enhance the DNA binding activity of AhR and increase its binding to AhRE at the IL-22 locus, which can induce transcription and promote IL-22 production (76). Lamas B et al. also found that CARD9-deficient mice had a reduced ability of the microbiota to metabolize tryptophan, resulting in reduced AhR activation of IL-22-producing T cells and ILCs, making IL-22 production reduced and more susceptible to colitis (77). A recent study in melanoma discovered that probiotic *Lactobacillus reuteri* was abundant in tumors compared to normal tissues. They released I3A, which can activate the AhR of CD8+ T cells to produce IFN-γ and enhance the efficacy of immune checkpoint inhibitor therapy (42). Overall, MTCs can act as ligands to activate the AhR of T cells and ILC3 and promote the production of cytokines such as IL-22 and IFN-γ, thus playing a protective role in carcinogenesis.

Pregnane X receptor (PXR) is another cellular receptor involved in the metabolic clearance of endogenous and exogenous substances, which is associated with a variety of cancers (144). Sári Z et al. also found that indolepropionic acid (IPA) can activate AhR and PXR, which can promote lymphocyte infiltration into tumors and enhance anti-tumor immune responses in breast cancer (78). Using PXR-or TLR4-deficient mouse models, Pondugula SR et al. showed that IPA produced by commensal intestinal bacteria activates the PXR of IECs, leading to down-regulation of TLR4 signaling. This results in the down-regulation of the proinflammatory cytokine TNF-α and the up-regulation of junctional protein-coding mRNAs to maintain the structure and function of the intestinal epithelial barrier (79).

### 3.2 MTCs indirectly regulate tumorigenesis by influencing host IDO1 expression and regulating immune cells

Both host tryptophan catabolites and MTCs such as indoles play important roles in the occurrence and development of host cancers. Indoleamine 2, 3-dioxygenase 1 (IDO1), is a rate-limiting enzyme of tryptophan catabolism, that is mainly activated in tumor cells, stromal cells, and antigen-presenting cells (145, 146). There is evidence that the host immunosuppressive effect on cancer is achieved in part by the conversion of tryptophan to kynurenine using IDO1 (147, 148). Change in tryptophan catabolism plays an important role in the occurrence of inflammation-driven cancers such as colon cancer (149). On the one hand, an enhanced kynurenine pathway can inhibit antitumor immune responses and promotes tumor growth (150). On the other hand, changes in the host intestinal microbiota lead to a decrease in the amount of tryptophan metabolized through the microbial indole pathway, a decrease in the availability of indole, which plays a protective role in the host, and aggravation of the host inflammatory response (151). Exacerbation of this inflammatory state can further increase IDO1 activity, which leads to apoptosis of T cells. At the same time, the cell-cycle of activated T cells is arrested due to the depletion of tryptophan. Changes in T cell function promote tumor proliferation *in situ* and distant metastasis (80–82). It has also been reported that tryptophan metabolism can play a pro-tumor role through the IDO-expressing DCs mediating the enhancement of Treg cell function and T cell anergy, forming an immunosuppressive microenvironment (83). In tumor-bearing mice, IDO-expressing DCs up-regulate immunosuppressive cytokines by activating Treg cells and inducing the recruitment of MDSCs (84, 85). Studies in human cells have also shown that Treg production and proliferation of Treg cells are associated with IDO-expressing DCs (86).

There is evidence that, in addition to preventing chronic inflammation, IDO1 inhibitors can also maintain homeostasis in the intestinal environment by altering the host microbial composition, resulting in increased production of the protective microbial-derived indole (152–154). IDO1 expression has been detected in a variety of cancers, including colorectal cancer, along with high kynurenine and low indole content (87, 88). It has also been shown that lymph node metastasis in patients with gastric adenocarcinoma (35) and endometrial cancer (39), as well as liver metastasis in patients with colorectal cancer (155), can be partially attributed to IDO expression. Therefore, promoting the growth of tryptophan-metabolizing bacteria such as *Lactobacillus* or increasing the production of indole, while reducing the utilization of tryptophan by host cancer cells, may be a new research direction for the inhibition of tumor growth.

### 3.3 MTCs depend on immune cell-derived MPO and epigenetic modifications to influence anti-tumor immunity

Metastatic pancreatic ductal adenocarcinoma (mPDAC) is a highly prevalent disease for which chemotherapy is considered the standard of care. However, less than half of patients respond to treatment (156). Recently, Tintelnot J et al. collected fecal samples from 30 mPDAC patients before the start of chemotherapy for shotgun metagenomic sequencing and metabolomic screening. They found that patients who responded to treatment had higher levels of 3-IAA and an increased number of *Bacteroides fragilis* and *Bacteroides thetaiotaomicron*. Combined experiments using loss of function and gain of function both *in vivo* and *in vitro* showed that microbial-derived 3-IAA relies on myeloperoxidase (MPO) to enhance the efficacy of the chemotherapeutic agent FIRINOX in the treatment of mPDAC. Myeloperoxidase oxidizes 3-IAA, which, through a series of enzymatic reactions, leads to ROS accumulation *in vivo* and down-regulation of tumor cell autophagy, thereby inhibiting tumor cell proliferation. 3-IAA makes even chemotherapy-resistant PDAC sensitive to treatment (36). In contrast, Alexeev E.E et al., using mouse colitis models, demonstrated that microbial-derived indole can inhibit the activity of MPO in polymorphonuclear leukocytes (PMN) that accumulate at inflammatory sites, thereby preventing inflammation and reducing bystander tissue damage (90).

In addition, Zhang et al. recently found that indole-3-lactic acid, a metabolite of *Lactobacillus plantarum L168*, could ameliorate intestinal dysbiosis and inhibit tumor growth in a mouse model of colorectal cancer. Mechanistically, the inhibition of tumor growth by indole-3-lactic acid is mainly related to epigenetics. Firstly, it can promote IL-12a production in DCs by enhancing H3K27ac binding at the IL-12a enhancer regions, to promote anti-tumor immunity of CD8+ T cells. Secondly, indole-3-lactic acid can also enhance the function of tumor-infiltrating CD8+ T cells by changing chromatin accessibility (89).

In conclusion, we reviewed that MTCs can act on immune cells, such as Th17 cells, Treg cells, CD8+ T cells, ILC3, and DCs, by activating AhR and PXR, affecting IDO and MPO activity or epigenetic modification, affecting cytokine production and tumor immune infiltration, which can inhibit the occurrence and metastasis of colorectal, breast, gastric, and endometrial cancers. MTCs can also act on PXR of IECs to maintain the intestinal epithelial barrier by down-regulating the TLR4 signaling pathway. In addition, MTCs can enhance the effect of immunotherapy for melanoma and chemotherapy for PDAC. However, while most studies have shown the tumor suppressive effect of MTCs, a recent study showed the opposite result. Their study indicated that dietary tryptophan or indoles could activate the AhR of tumor-associated macrophages to reduce the accumulation of TNFα+IFNγ+CD8+ T cells, impairing the anti-tumor immune response in PDAC (157). Moreover, as mentioned above, the effect of MTCs on MPO is also opposite in different tumors (36, 90). This suggests that more observational and experimental studies are needed in the future to further explore the mechanism of MTCs affecting tumor progression, and more attention should be paid to heterogeneous tumors and the heterogeneity between different tumors.

## 4 PAs

PAs, including cadaverine, putrescine, spermine and spermidine, are small polycationic molecules that play a role in a variety of biological processes (9). Arginase 1, which syntheses arginine to ornithine, ornithine decarboxylase, which converts ornithine to putrescine, and a series of enzymes required in the interconversion of spermine, spermidine, and putrescine are involved in the anabolic process of PAs (5). In addition, microbiota at the lower gut level can also use amino acid decarboxylase to metabolize amino acids to produce microbiota-derived PAs (5, 9). PAs are at high levels in tumor cells and can affect the tumor microenvironment and prevent the activation of immune cells, resulting in carcinogenic effects.

### 4.1 PAs influence anti-tumor immune response by altering the immunosuppressive tumor microenvironment

Intracellular PAs can be from the diet, the intestinal microbiota, and the host itself, and their contents in the body are regulated by various pathways such as catabolism, biosynthesis, export and absorption (158). In terms of immunity, as metabolites related to microflora and carcinogenesis, PAs have important effects on the activation of B and T cells (91). PAs have also been implicated in the promotion of T-cell proliferation and induction of cytotoxic activity (92, 93). Increased PA levels can lead to immunosuppression of the tumor microenvironment (94), as well as decreased expression of adhesion molecules such as LFA-1 and CD44 (95, 96), and production of cytokines such as TNF and IFN-γ (97, 98), weakening with anti-tumor immune responses. Studies have shown that PAs are found at high levels in many cancers, including brain tumors, neuroblastoma, endometrial cancer, breast cancer and colorectal cancer (159). In these cancers, levels of PAs are higher in tumor cells than in normal cells (160). *In vivo*, there are not only oncogenes such as RAS and MYC that promote polyamine synthesis (161, 162), but also polyamine transport systems (PTS) that can increase cellular uptake of microbiota-derived PAs (163).

Given the role of PAs in the occurrence and development of tumors, the synthesis and transport of PAs have become new targets for cancer treatment, and polyamine blockade therapy (PBT) has become a new research hotspot. The rate-limiting enzyme of PA biosynthesis *in vivo* is ornithine decarboxylase (ODC). And α-difluoromethylornithine (DFMO) can inhibit its activity (99). However, studies have found that although DFMO alone can reduce PA biosynthesis in tumor cells, tumor cells will also increase the uptake of PAs from diet and intestinal microbiota through a compensatory increase in PTS activity. Finally, it is difficult to achieve a good therapeutic effect, because the concentration of PAs in tumor cells is not significantly reduced (164, 165). Encouragingly, a novel polyamine transport inhibitor, trimer PTI, has emerged. PBT focuses on the combined application of DFMO and PTI to enhance the effect (99). To enhance the effect of polyamine-targeted cancer therapy by simultaneously reducing PAs synthesis of cancer cells and reducing their ability to uptake exogenous PAs such as microbiota-derived PAs (166).

The efficacy of PBT has been demonstrated in mice with colon cancer. PBT treatment reduced tumor growth in mice and increased levels of cytokines such as IL-10, MCP-1, and IFN-γ, which are associated with enhanced immune activity in tumors and tumor microenvironments. However, if CD4+ and CD8+ T cells were depleted in mice, the anti-tumor effect of PBT was lost. Further studies found that one of the mechanisms of PBT action is to increase the activity of cytotoxic T cells and reduce the tumor-infiltrating immunosuppressive myeloid cell population. Tumor cells can be directly killed by cytotoxic T cells, and tumor-infiltrating immunosuppressive myeloid cell populations such as MDSCs, M2 macrophages, and Treg cells can inhibit the activity of CTLs (99). Previous studies in melanoma have also shown that PBT can also impair immunosuppressive activity by reducing arginase activity in cells such as MDSCs, IL-4-induced macrophages, and tumor cells with elevated PA levels (167, 168). In addition, PAs released by MDSCs can also confer an immunosuppressive phenotype on DCs (169). It is concluded that PBT can enhance anti-tumor immune responses by affecting PA metabolism in tumor cells and a variety of cells that exert immunosuppressive effects. In summary, PBT can inhibit tumor growth through a variety of mechanisms.

In conclusion, we reviewed that high levels of PAs shape the immunosuppressive tumor microenvironments and promote a variety of tumors by acting on immune cells such as T cells, B cells, MDSCs, macrophages, DC. PBT targeting PA synthesis and transport can enhance the therapeutic efficacy of colon cancer, melanoma.

## 5 Hydrogen sulfide

Hydrogen sulfide is an endogenous gas in mammals that plays a signaling role (170). In the early 20th century, studies on microbiota have found that bacteria can produce hydrogen sulfide (171). Specifically, it is produced by anaerobic sulfate-reducing bacteria (SRB), such as *Desulfobacter, Enterobacter, Helicobacter pylori, Escherichia coli, Fusobacterium nucleatum, Staphylococcus aureus, Klebsiella, Salmonella thyphimurium* and various *Clostridium* species, which metabolize dietary sulfate in the colon (10, 172). Whether hydrogen sulfide is beneficial or harmful in the host depends on its concentration, and its effect on tumors is also two-sided.

### 5.1 Bacterial hydrogen sulfide is involved in tumorigenesis and development by regulating its defense mechanisms and host intestinal mucosal immunity

Some of the hydrogen sulfide produced by gut bacteria can first enter the host circulation as oxidation products and/or free hydrogen sulfide and then be reconverted to hydrogen sulfide in the host body (173). Hydrogen sulfide has a bell-shaped effect that is protective of the intestinal epithelium at low concentrations and harmful at high concentrations. On the one hand, bacterial hydrogen sulfide supports the metabolism of IECs, and on the other hand, in patients with colorectal cancer, higher than normal concentrations of hydrogen sulfide at the tumor site can disrupt intestinal mucosal immunity and promote cancer progression (26).

Several pieces of evidence suggest that a high abundance of SRB promotes diseases such as IBD and colorectal cancer (11). Figliuolo VR et al. inoculated SPF mice intragastrically with SRB and found that when SRB colonized the gut of SPF mice and produced hydrogen sulfide, the body had an inflammatory response and a series of changes in the composition and function of immune cells occurred. The numbers of CD11b+ leukocytes, B cells, CD8+ T cells and Treg cells significantly increased, suggesting that hydrogen sulfide can stimulate immune responses in wide types of immune cells. Increased Treg cells produce more IL-10, which in turn inhibits IL-2 production in lymph nodes and maintains a balanced immune response. There was also a sharp increase in Th17 cells and an increase in the production of the proinflammatory cytokines IFN-ɣ, IL-6, and IL-17A, suggesting that SRB can trigger Th17-mediated inflammatory responses. In addition, SRB further enhanced the inflammatory response in colitis mice (58).

*In vivo* and *in vitro*, bacteria-derived hydrogen sulfide can also upregulate their antioxidant defense mechanisms (101), allowing them resistant to host leukocyte-mediated killing to protect them from antibiotic damage (102). Studies *in vitro* and mouse abdominal sepsis models have shown that elevated levels of hydrogen sulfide increase the resistance of *Escherichia coli* and *Staphylococcus aureus* to immune-mediated killing. Conversely, inhibiting the production of hydrogen sulfide enhances the susceptibility of both bacteria to rapid killing by immune cells. This suggests that hydrogen sulfide produced by bacteria can protect itself by reducing the impact of the early host immune response, such as neutrophil-mediated elimination (102). *Fusobacterium nucleatum* is a known hydrogen sulfide-producing bacterium that is suspected to promote colorectal cancer. Based on the relative abundance of bacteria, Hale VL et al. used a microbiota metabolic simulation system to predict an increase in the abundance of *Fusobacterium nucleatum* in colon cancer samples and an increase in intestinal bacteria-derived hydrogen sulfide (27).

The role of colonic bacteria-derived hydrogen sulfide causing inflammation and tumors has been studied primarily in IBD (174). The increased concentration of luminal hydrogen sulfide disrupts the detoxification system of IECs, allowing local hydrogen sulfide to become cytotoxic and proinflammatory (170). Recurrent episodes of colonic or rectal inflammation increase the incidence of inflammation-related precancerous lesions and tumors (175). By reducing the disulfide bonds between mucin 2, microbiota-derived hydrogen sulfide can disrupt the homeostasis of the body’s intestinal mucosa, leading to epithelial cell damage and the onset of various inflammation-related intestinal diseases (28). Studies have found that hydrogen sulfide promotes the development of colorectal cancer mainly by inducing excessive proliferation of colonic mucosa, mediated by mitogen-activated protein kinase (MAPK, originally known as ERK) (100, 176) and inducing proliferation and differentiation of IECs through the RAS-MEK-ERK pathway (177).

Contrary to the pro-inflammatory and pro-cancer effects mentioned above, Ji et al. revealed the anti-tumor effects of bacterial hydrogen sulfide, recently. Metabolomics analysis of fecal metabolites in C57BL/6J mice fed with the methionine restricted (MR) diet revealed reduced hydrogen sulfide production in feces induced by the MR. Furthermore, it was found that MR caused a smaller proportion of circulating T cells and a larger tumor weight in mice. These indicated that the MR diet can inhibit the production of hydrogen sulfide by intestinal bacteria and inhibit the activation of T cells, which directly leads to defective anti-tumor immunity. Overnight culture of activated human peripheral blood mononuclear cells in an MR medium further revealed that hydrogen sulfide may enhance the survival/activity of immune cells by increasing cysteine sulfhydration and glycolysis (103). Encouragingly, Qiu et al. recently proposed a new tumor treatment method, which uses injectable chondroitin sulfate (ChS) hydrogel loaded with SRB (SRB@ChS Gel) to continuously produce hydrogen sulfide in tumor tissues, which can activate systemic anti-tumor immunity and suppress distant and recurrent tumor growth, as well as lung metastasis, with minimal side effects (178).

In conclusion, we reviewed that hydrogen sulfide exerts beneficial or harmful effects in the host highly dependent on its concentration, and its effects on tumors are also two-sided. Hydrogen sulfide can affect the function of host immune cells, and regulate the antioxidant defense mechanism of bacteria and the intestinal mucosal immunity of the host, to promote or inhibit host tumors at different concentrations. High concentrations of hydrogen sulfide *in vivo* can affect host immune cells, such as Treg cells, Th17 cells, and IECs, and up-regulate the antioxidant defense mechanism of bacteria to promote tumors. While at therapeutic concentrations, it can activate an anti-tumor immune response to inhibit tumor genesis and metastasis. Therefore, in the future, the dual effects of bacterial hydrogen sulfide on host anti-tumor immunity should be further explored, and further exploration of the boundary between high and low concentrations will facilitate SRB-targeted tumor therapy.

## 6 Secondary bile acids

Cholesterol is a class of lipids that are catabolic in the liver into primary bile acids, such as chenodeoxycholic acid (CDCA) and cholic acid (CA) (12), The secondary bile acids, lithocholic acid (LCA) and deoxycholic acid (DCA), are unbound forms of CDCA and CA, respectively, which are metabolized by the intestinal microbiota (9). Anaerobic bacteria such as *Clostridium* are responsible for bile acid conversion (179). Most of the bacteria that produce secondary bile acids come from *Bacteroides, Lactobacillus, Bifidobacterium, Clostridium (clusters XIVa* and *XI)* and *Eubacterium* (7, 9, 12). Bile acids have both anti-inflammatory and pro-inflammatory effects when present at low and high concentrations, respectively, by activating different receptors. Acting as ligands, secondary bile acids bind to immune cells to exert anti-inflammatory and detoxification effects, thereby protecting the body from inflammation and tumors.

### 6.1 Secondary bile acids activate various transmembrane and nuclear receptors to regulate inflammation-driven cancer by regulating mucosal immunity

Studies have shown that secondary bile acids produced by intestinal microbial metabolism can directly act on various nuclear and transmembrane receptors expressed in IECs or immune cells to be involved in the regulation of the intestinal mucosal barrier and play an important role in the occurrence and development of colorectal cancer (29).

As two classical receptors involved in bile acid metabolism, the G protein-coupled receptor TGR5 and the nuclear receptor farnesoid X receptor (FXR) on the cell surface play anti-inflammatory roles in the intestinal mucosal immune system and reduce inflammation-driven cancer. TGR5 is highly expressed in IECs and a variety of hematopoietic cell lines, especially macrophages and monocytes. It has a high affinity for DCA and LCA and is easily activated by them (180, 181). *In vivo* and *in vitro* studies have shown that bile acids can activate TGR5 in bone- marrow-derived macrophages, human peripheral blood monocytes, mouse peritoneal macrophages, and bone-marrow-derived DCs, to block the activation of NLRP3 inflammasome by epigenetic modification, which is phosphorylation on a single residue, Ser 291 and ubiquitylation through the TGR5-cAMP-PKA axis (104). In colitis mouse models, bile acids can effectively limit M1 macrophages to secret proinflammatory cytokines such as TNF-α, IFN-γ, IL-6, and IL-12, and increase the binding of macrophages to IL-10 gene promoter and differentiation into IL-10 secreting M2 macrophages. dependent on TGR5. It is IL-10 dependent and promotes Treg cell recruitment to inflamed colonic tissues (105). Bile acids can also activate FXR and alter its interaction with transcriptional cofactors, leading to altered post-translational modifications of FXR and histones, which effectively regulate the expression of target genes (182). Massafra V et al. found in mice models of colitis that obeticholic acid (OCA) can activate FXR of splenic DCs, allowing them to persist. Subsequently, this results in increased plasma IL-10 levels, decreased colonic Madcam1 expression, and increased Ccl25 expression. These changes alter the chemotactic environment at sites of inflammation in the colon and induce an increase in Tregs, which exhibit anti-inflammatory effects (106). DCA can be converted to 3β-hydroxydeoxycholic acid (isoDCA) *in vivo* (183). Campbell C et al. designed minimal microbial consortia containing engineered *Bacteroides* strains. Using cell co-culture and mouse experiments, they showed that isoDCA can act on FXR of DCs to restrict its activity, which caused reduced transcription of genes involved in antigen processing and presentation, detection and transduction of proinflammatory factors, and downstream of interferon signaling. Next, the extrathymic differentiation of colonic RORγt+ Treg cells was induced in a CNS 1-dependent manner, presenting an anti-inflammatory state. However, isoDCA had no substantial effect on Th17 cells (184). Furthermore, activation of FXR has been shown to restrict the expression of inflammatory cytokines such as IL-1β and IL-6, and chemokines such as CCL2 in mouse colitis models and human CD14+ monocytes and DCs *in vitro* (107). Activation of FXR on macrophages and DCs by secondary bile acids can reduce the expression of TLR4-dependent proinflammatory cytokine and inhibit activation of the NLRP3 inflammasome (108, 109).

Unlike activation of FXR and TGR5, which exert an anti-inflammatory effect, bile acids have pro-inflammatory effects at high concentrations due to membrane disruption and cytotoxicity (185). For IECs, at low concentrations, secondary bile acids can regulate epithelial cell integrity and microbial composition by binding to FXR (110). While at high concentrations, secondary bile acids metabolized by microbial metabolism, especially LCA, can activate three nuclear receptors including PXR, Vitamin D receptor (VDR), and constitutive androstane receptor (CAR), and act as intrinsic regulators of IECs function under dynamic equilibrium conditions, playing roles in promoting bile acids detoxification and protecting tissues from bile acids damage (185, 186). Mucosal tissue biopsies from patients with IBD showed reduced expression of PXR, VDR, and CAR target genes. It can be seen that PXR, VDR and CAR have a role in protecting the intestine from disease invasion (187, 188). Normally, activated PXR promotes the expression of TGF-β and limits the expression of TNF-α, CCL20, CCL5 and IL-8 in IECs (111, 112). It can also play a protective role by reducing the stability of TLR4 mRNA, reducing TLR4 signaling, and inhibiting the production of TLR4-dependent proinflammatory cytokines in IECs (113). PXR and its related heterogenic sensitive nuclear receptor, CAR, also can reduce the toxicity of bile acids and promote their elimination from the body by inhibiting NF-κB activation and drug metabolization enzyme (DME) expression (111, 114). As with the activation of PXR, LCA can also activate VDR in IECs and induce CYP3A-mediated bile acid detoxification (189). LCA-dependent VDR activation suppressed the expression of proinflammatory cytokines in IECs during experimental colitis, and LCA had no anti-inflammatory effect in VDR-deficient mice (115, 116). In addition, VDR is also involved in the regulation of immune cell function, which can promote the differentiation of Treg cells, inhibit the activation of monocyte-derived macrophages, inhibit the maturation of DCs, and reduce the secretion of proinflammatory cytokines Th1 and Th17 (57). In summary, secondary bile acids metabolized by microbial metabolism can play a protective role in the occurrence and development of host chronic intestinal inflammation and inflammation-driven cancer by activating the above three nuclear receptors.

### 6.2 Secondary bile acids regulate liver cancer and cancer liver metastasis through NKT cells

The liver is an immune organ composed of a large number of immune cells. Intestinal bacterial components and metabolites enter the liver through the portal vein. Changes in intestinal bacterial composition or biological dysregulation may affect the function of immune cells in the liver and cause liver carcinogenesis (190). Liver metastases are also the most common metastatic cancer in the body (191). At the same time, the changes in intestinal microbiota may affect the function of immune cells in the liver. Using one primary liver cancer and three liver metastasis mouse models, Ma et al. found that alterations in gut commensal bacteria could specifically affect intrahepatic tumor progression (21). Further studies showed that the accumulated liver NKT cells played an important role in this process. Activated NKT cells could produce more IFN-γ, enhance liver-selective tumor suppression, and inhibit liver cancer growth (21).

The survival and accumulation of NKT cells in the liver have been reported to be mediated by the chemokine receptor CXCR6, whose sole ligand is CXCL16. In the liver, CXCL16 which is produced primarily by liver sinusoidal endothelial cells (LSECs), is the first barrier for blood to enter the liver (192). *In vitro* experiments demonstrated that secondary bile acids could inhibit CXCL16 expression, while primary bile acids had the opposite effect. In mice, feeding with primary bile acids or treatment with vancomycin, which eliminates bile acid-converting bacteria, induced liver NKT cell accumulation and inhibited liver tumor growth. However, feeding secondary bile acids or colonizing bile acid-metabolizing bacteria reversed the accumulation of NKT cells and their suppression of liver tumor growth (21). This has also been demonstrated by studies of the genomes of human liver cancer patients (117). In conclusion, the intestinal microbiome can use bile acids as messengers to regulate the level of CXCL16 and influence the growth of liver cancer by controlling the aggregation of CXCR6+ liver NKT cells.

In conclusion, we reviewed that the effects of secondary bile acids *in vivo* are concentration-dependent. At low concentrations, secondary bile acids can restrict the activation of NLRP3 by acting on TGR5 and FXR in macrophages, monocytes and DCs, phosphorylating and ubiquitinating NLRP3, which can regulate the production of cytokines and induce the extrathymic differentiation of Treg cells to exert anti-inflammatory effects. At high concentrations, secondary bile acids are toxic and can activate PXR, VDR and CAR in IECs to play a detoxification effect. In addition, secondary bile acids can also inhibit the activation of NKT cells by inhibiting the expression of CXCL16 in liver sinusoidal endothelial cells, and promote liver cancer and cancer liver metastasis.

## 7 Host-diet intake influences the intestinal microbial composition and metabolite generated

Since most intestinal microbial metabolites are produced by microbial metabolism of host dietary components, host-diet intake plays a significant role in intestinal microbial composition and metabolite production. Prebiotics have a beneficial effect on the intestinal microbiota. Prebiotics typically refer to fermentable carbohydrates in food that cannot be digested, such as inulin and oligosaccharides (193, 194). *In vitro* and *in vivo*, studies have confirmed that consumption of prebiotics can promote the growth of potentially protective bacteria such as *bifidobacteria* and certain *Lactobacilli*, and/or inhibit the pathobionts such as *Clostridium spec., E. coli, Campylobacter jejuni, Enterobacterium spec., Salmonella enteritidis* or *S. typhimurium*. These effects have been observed in humans, mice, and piglets, indicating a protective effect (195). For example, a recent study in non-small cell lung cancers (NSCLCs) showed that ginseng polysaccharides, acting as a prebiotic, can enhance the presence of *Parabacteroides distasonis* and *Bacteroides vulgatus*. This, in turn, activates CD8+ T cells and suppresses Treg cells by increasing the microbial metabolite valeric acid and reducing the levels of L-β Kyn and Kyn/Trp ratios. These findings aim to improve the efficacy of anti-PD-L1/PD-L1 immunotherapy in combating tumors (196). Resistant starch type 4 (RS4), which is chemically modified to achieve undigestibility, is another prebiotic. Martinez I et al. examined human fecal samples with pyrosequencing of 16S rRNA tags and found that consumption of RS4 resulted in a significant decrease in *Firmicutes*, as well as increases in *Bacteroidetes* and *Actinobacteria* (197). Liu et al. also found increased cecal butyrate concentration in RS4-fed mice. And butyrate can increase the tri-methylation of lysine 27 on histone 3 (H3K27me3) of NF-κB1 promoter in colonic epithelial cells, leading to inhibiting the expression of NF-κB1 and its downstream effector CCL2. An inverse correlation between H3K27me3 enrichment and a concentration-dependent downregulation of NF-κB1 was also found in human colonic epithelial cells treated with sodium butyrate. These results indicated that the intake of prebiotic RS4 could alter the colonic microbiota and increase the content of butyrate. Then, it can modify the promoter region of NF-κB1 in colon epithelial cells by methylation and inhibit the pro-inflammatory NF-κB pathway, exhibiting anti-inflammatory effects (198).

On the other hand, the intestinal microbiota interacts with the host in a dynamic equilibrium relationship. Once its composition becomes unbalanced, a condition called dysbiosis occurs, resulting in abnormal metabolite production that harms the overall health of the host. For instance, Fujita K et al. have suggested that a High-Fat Diet (HFD) can cause intestinal dysbiosis in prostate cancer, leading to an increase in some harmful bacteria such as *Bacteroides massiliensis*, *Streptococcus, Bacteroides species, Rikenellaceae, Alistipes* and *Lachnospira*. Intestinal bacterial metabolites, such as SCFAs and phospholipids, enter the systemic circulation, thereby promoting the growth of prostate cancer (199). Similarly, a study by Yang et al. in colorectal cancer demonstrated the dangers of a HFD. They found that high-fat feeding increased the abundance of pathobionts *Alistipes* sp. *Marseille-P5997* and *Alistipes* sp. *5CPEGH6* while depleting probiotics *Parabacteroides distasonis* in mice. Furthermore, the intestinal metabolite lysophosphatidic acid was found to be elevated. The study concluded that a HFD drives the development of colorectal tumors by inducing intestinal microbial dysbiosis, metabolomic dysregulation with elevated lysophosphatidic acid and intestinal barrier dysfunction (200). Zhang et al. conducted research that found a high-cholesterol diet to increase the abundance of *Mucispirillum, Desulfovibrio, Anaerotruncus* and *Desulfovibrionaceae* in mice, while decreasing *Bifidobacterium* and *Bacteroides*. Additionally, the high-cholesterol diet caused changes in the metabolites produced by intestinal bacteria, including an increase in taurocholic acid and a decrease in 3-indolyl propionic acid. All these changes drive the development of Non-alcoholic fatty liver disease (NAFLD)- associated hepatocellular carcinoma (HCC) in mice (201).

In summary, host-diet intake affects host health in both directions by influencing the production of intestinal microbiota and their metabolites. The consumption of prebiotics can promote the growth of probiotics and inhibit the growth of pathogenic bacteria, thereby protecting health and aiding in tumor treatment. Conversely, a high intake of fat, cholesterol and similar substances can lead to a decrease in probiotics and an increase in harmful bacteria, causing dysbiosis and resulting in the occurrence and development of tumors and other diseases.

Bacteria are mainly classified as symbiotic bacteria that constitute intestinal symbionts, and pathogenic bacteria that are foreign and harmful to health, based on their relationship with the host. The vast majority of intestinal symbionts are mutualistic that are beneficial to health or commensals that provide no obvious benefit to the host. However, under certain circumstances, some of the indigenous bacteria can promote disease and are commonly referred to as pathobionts (202). Symbiotic bacteria in the host can promote immune homeostasis, immune responses and prevent pathogen colonization. Once the environmental and genetic factors increase the perturbation of the structure of intestinal flora, it may lead to the emergence of pathobionts and infection of pathogenic bacteria, causing the occurrence of inflammation and cancer (202). Antibiotics are the conventional method for the treatment of pathogenic inflammation. However, antimicrobial resistance has become a great challenge (203). There have been many new attempts to *alleviate* pathogenic inflammation, such as probiotics and vaccine strategies.

Probiotics are defined as “viable microorganisms, sufficient amounts of which reach the intestine in an active state and thus exert positive health effect” (195). Currently, the most popular probiotic strains are *Lactobacillus* spp. and *Bifidobacterium* spp. and probiotics are emerging research directions for reducing pathogenic inflammation. For example, *Helicobacter pylori* (*H. pylori*) is considered to be one of the most important pathogenic bacteria causing atrophic gastritis and gastric cancer (204). Probiotics can combat *H. pylori* infection by competing with H. pylori for binding sites on gastric epithelial cells, strengthening the mucosal barrier, and secreting bactericidal organic acids such as lactic acid (205). *Lactobacillus reuteri* (206), *Lactobacillus acidophilus* (207), *Lactobacillus bulgaricus* (207) *Lactobacillus rhamnosus* (208), *Lactobacillus acidophilus* (208) and *Lactobacillus plantarum* (pH3A) (209) have been shown to reduce mucosal H. pylori density and significantly improve gastric mucosal inflammation. Although, probiotics can help to reduce pathogenic inflammation. traditional and widely used probiotics are not disease-specific and have limited effect on disease improvement. Surprisingly, the development of next-generation sequencing technology and bioinformatics technique platforms makes it possible to identify the next generation probiotics (NGP), such as *Faecalibacterium prausnitzii*, *Akkermansia muciniphila* and *Bacteroides fragilis* (210). In addition, advances in metabolic engineering and synthetic biology have made it possible to design engineering probiotics with desirable properties and functions that target specific tissues and cells and *Escherichia coli* is a hot spot for modification (211).

Vaccines are another emerging research direction to reduce pathogenic inflammation (212). For example, the *Shigella* is a pathogenic bacteria agent of severe diarrhea and dysentery. Gerke C et al. designed a vaccine (1790GAHB) against *Shigella sonnei* using the generalized membrane antigen module (GMMA) technology using the O-antigen (OAg) fraction of LPS as the active fraction (213), which has been proved to be effective and safe by phase II clinical trials (214). Riddle MS et al. conjugated the polysaccharide component of the O-antigen of *S. flexneri 2a* to exotoxin protein A of *Pseudomonas aeruginosa* (EPA) and developed a novel bioconjugate vaccine (Flexyn2a) against *S. flexneri 2a* (215), which have been proved promising (216). Although great achievements have been made in developing vaccine strategies, we still face many problems. First, vaccines against many important pathogens are still lacking. Second, much research on vaccines against pathogens is at an early stage and faces obstacles such as funding constraints. Third, the side effects of the vaccines that have been developed have not been fully revealed, and the safety profile needs to be better elucidated.

## 8 Discussion

The complexity and breadth of the interactions between microbiota and their hosts have been demonstrated in a long history of scientific research. As chemical messengers between microbiota and host, microbial metabolites play an important role in human pathophysiological processes, have a profound impact on the host immune system, and are closely related to the occurrence and development of human diseases such as tumors. However, there are still many gaps in the impact of microbiota and its metabolites on tumors.

First, there is significant individual variability in microbial populations at the genus, species, and strain levels (4), which reflects host-specific dynamics due to host lifestyle, physiology, or genetic differences (217). There are inter-subject variations in the response to interventions targeting the microbiome (197). Therefore, personalized medicine must be developed, which can tailor medical decisions for individual patients or specific patient groups, to maximize the curative effect and avoid ineffective treatment (218).

Second, although a variety of microbial metabolites have been identified, their functions are not fully understood, and the molecular mechanisms involved in some interactions are still unclear. On the other hand, there are still many new microbial metabolites that have not been discovered. For example, metabolic reprogramming and epigenetic modifications of tumor cells and immune cells are some of the important features of cancer (219). The intestinal microbiota can influence host epigenetic modifications by producing metabolites, which can influence cancer progression through epigenetic pathways such as methylation, acetylation, and chromatin accessibility (220). However, many mechanisms by which intestinal microbial metabolites contribute to cancer progression through epigenetic modifications on immune cells and IECs remain unexplored, such as the effect of metabolites on DNA methylation. Sobhani I et al. transplanted feces from health people and sporadic colon cancer patients into germ-free mice and found that the colon cancer-associated microbiota, with a higher abundance of *Parvimonas* and *Parasutterella*, induced more hypermethylated genes, such as SFRP1,2,3, PENK, NPY, ALX4, SEPT9, and WIF1, in the colonic mucosa of mice than the microbiota receptors of healthy controls. And the concentration of SCFAs in feces was significantly reduced (221). This suggests that microbiota-derived SCFAs may be associated with host gene DNA methylation/demethylation. However, the effect of SCFAs on host epigenetic modification is mainly focused on inhibiting histone deacetylation by inhibiting HDAC, while their effect on DNA methylation/demethylation is still unclear. These gaps in knowledge need to be further explored to truly use microbiota and their metabolites as targets for cancer therapy. The development of metabolome is expected to provide help for a more systematic and comprehensive study of the effects of known microbial metabolites on the occurrence and development of tumors and the discovery of new microbial metabolites. Testing for metabolites is relatively convenient, and inexpensive, and may reveal abnormalities before patients have overt clinical symptoms. An in-depth understanding of the role of microbial metabolites in tumorigenesis and development may identify potential tumor biomarkers and provide opportunities to find new directions for cancer prevention and treatment.

In summary, although many studies have shown the correlation between microbial metabolites as well as their regulation of immune response and the occurrence and development of tumors, there are still many unknown biological mechanisms waiting for researchers to explore. A large amount of basic research and clinical data are needed to support the use of SCFA, indole acid derivatives, PBT, SRB, secondary bile acids, probiotics or antibiotic vaccination for cancer treatment and there is a certain distance from the real application in clinical practice. In the future, the microbiome and its metabolites will remain one of the most promising fields in cancer research and are highly likely to be effective targets for cancer prevention and treatment.

## Author contributions

JL: Writing – original draft, Writing – review & editing. RT: Writing – review & editing. CS: Writing – review & editing. YG: Writing – review & editing. LD: Writing – review & editing. YL: Writing – review & editing. XS: Writing – review & editing.
